# No genetic causal relationship between acne and prostate cancer through Mendelian randomization combined with meta-analysis

**DOI:** 10.3389/fonc.2024.1460467

**Published:** 2024-09-19

**Authors:** Mingyang He, Xin Wang, Dongbin Wang, Yaxuan Wang, Jinchun Qi, Jianghua Jia, Ming Zhang, Qingsong Meng, Bowen Yan, Heyang Guo, Changbao Qu

**Affiliations:** Department of Urology, The Second Hospital of Hebei Medical University, Shijiazhuang, Hebei, China

**Keywords:** prostate cancer, Mendelian randomization analysis, meta-analysis, morbidity hazard factor, acne

## Abstract

**Background:**

Previous observational studies regarding the relationship between acne and prostate cancer have reported inconsistent results. As such studies are prone to biases, we conducted this Mendelian randomization (MR) analysis to better explore the causal association between acne and prostate cancer.

**Methods:**

The genetic data for assessing acne were acquired from the largest genome-wide association study (GWAS) of acne by far, and the genetic data for assessing prostate cancer were acquired from the FinnGen consortium, UK Biobank, European Bioinformatics Institute, and IEU OpenGWAS project. We performed two-sample MR analyses using data from these GWASs followed by a meta-analysis to provide an overall evaluation. The primary MR methods used included inverse variance weighted, MR-Egger, and weighted median. Leave-one-out sensitivity tests, Cochran’s Q tests, and MR-Egger intercept tests were used to bolster the robustness of the MR results.

**Results:**

Through MR combined with meta-analysis, our study found no genetic causal relationship between acne and prostate cancer (*p*=0.378; odds ratio=0.985; 95% confidence interval, 0.954–1.018). Sensitivity tests ensured the robustness of this result.

**Conclusion:**

Acne should not be considered as a morbidity hazard factor for prostate cancer.

## 1 Introduction

Prostate cancer (PC) is the second-most frequent malignancy and the sixth major cause of cancer-related fatalities in men globally (1). The prostate cancer incidence rate has risen by 3% per year from 2014 to 2019 in the USA (2), and it is predicted that there will be approximately 2.3 million new cases and 740,000 deaths from PC globally in 2040 (3). Therefore, it is important to detect suspected at-risk patients to reduce the PC incidence and mortality rates. Acne is a very common chronic inflammatory disease of the skin. According to statistics, acne has become the eighth-most prevalent disease worldwide, affecting approximately 9% of the global population (4). The pathogenesis of acne involves multiple factors such as inflammation caused by *Propionbacterium acnes* and the hormonal influence (5). *P. acnes*, as an opportunistic pathogen, plays a likely underestimated role in the development of other human diseases such as degenerative disk, prostate disease, and atherosclerosis (6, 7).

Because acne is a proxy for androgen status (8) and *P. acnes* is reported to be associated with prostatic inflammation and carcinogenesis (9, 10), there have been studies examining the association between acne and the risk of prostate cancer. However, these previous studies exploring the association between acne and prostate cancer reported inconsistent results. A large prospective population-based cohort (11) indicated that acne was associated with an increased risk for prostate cancer. Another study (12) suggested that individuals with acne in adolescence have a higher risk of adult prostate cancer mortality. One study suggested a lack of acne during adolescence reduce high-grade prostate cancer at adulthood (13). Some other studies (14, 15) showed no associations between acne and the risk of prostate cancer. Additionally, one case–control study (16) reported a negative association between acne‐related facial scarring and later prostate cancer. These observational epidemiological studies are limited in assessing causality due to confounding and reverse causation. Therefore, we hope this question will be resolved with our proposed method.

Mendelian randomization (MR) is a genetics-based instrumental variable approach that relies on the random and fixed assignment of genetic variants at conception to estimate the causal effect size of genetically predicted exposures on an outcome. Compared with traditional observational studies, MR is less susceptible to confounding or reverse causation (17). In addition, a meta-analysis is a statistical method that combines the results of different analyses about the same topic, which can solve conflicts somewhat. It can help to integrate the MR results from different populations, thus drawing a reasonable conclusion. In our study, we use publicly available genome-wide association study (GWAS) data setting genetic variations associated with acne as instrumental variables (IVs), then explored the causal relationship between acne and PC through two-sample MR analyses, followed by a meta-analysis to provide an overall evaluation.

## 2 Materials and methods

### 2.1 Study design overview

**Figure 1:**
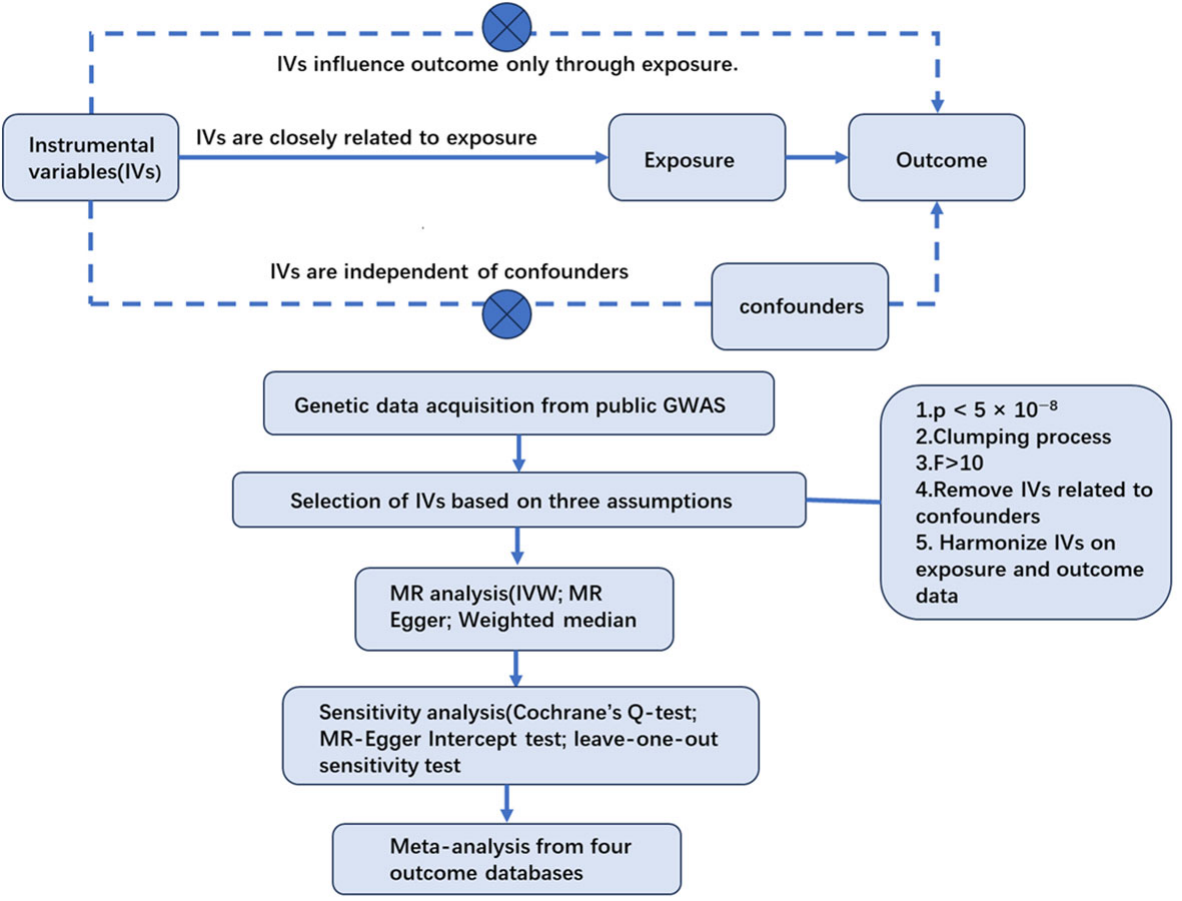
Flowchart of the MR study.

This study considered acne as the exposure and PC as the outcome. The single nucleotide polymorphisms (SNPs) closely related to acne were regarded as IVs. After the selection of IVs, the causal association between acne and PC was explored by two-sample Mendelian randomization analyses. Then, sensitivity tests were carried out to ensure the results were robust and accurate. Finally a meta-analysis was conducted to integrate MR results from different outcome databases (**Figure 1**).

The IVs for MR analysis must meet three basic assumptions (18): (1) the correlation assumption—the IVs are closely related to exposure; (2) the independence assumption —the IVs are independent of confounding factors between exposure and outcome; and (3) the excluding restriction assumption—the IVs influence outcome only through their effects on exposure and not through other casual pathways.

We performed these analyses using the “TwoSampleMR” package and “meta” package in Rstudio (version 4.3.3.).

### 2.2 Data sources

The genetic data for assessing acne were acquired from the largest GWAS meta-analysis of acne by far, which included 20,165 European ancestry cases and 595,231 European ancestry controls (19). The definitions of acne in these cohorts varied across clinical assessments, electronic health records, and self-report questionnaires. The GWAS summary statistics are available in the GWAS catalogue (www.ebi.ac.uk/gwas) (accession number GCST90092000). The genetic data for assessing PC were acquired from four eminent databases: the FinnGen consortium (N=146465), UK Biobank (N=463010), the European Bioinformatics Institute (N=208768), and the IEU OpenGWAS project (N=140254). The detailed information regarding these genetic data are listed in **Table 1**.

**Table 1:** Description of the data sources of outcome.

Database	Year	Trait	Population	Number of cases	Number of controls	Number of SNPs
FinnGen consortium	2023	Malignant neoplasm of prostate	European	15,199	131,266	21,283,394
IEU OpenGWAS project	2018	Prostate cancer	European	79,148	61,106	20,346,368
UK Biobank	2018	C61 Malignant neoplasm of prostate	European	3,436	459,574	9,851,867
European Bioinformatics Institute	2021	Prostate carcinoma	European	3,221	205,547	11,842,647

### 2.3 Selection of IVs

To satisfy the three basic assumptions, we conducted the selection of IVs using the following steps. (1) We used the threshold (p < 5 × 10^−8^) to select SNPs closely related to acne. (2) We eliminated the linkage disequilibrium (LD) between chosen IVs by controlling parameters of r2 <0.001 and a clumping window of >10,000 kb. LD refers to the phenomenon that genetic variants physically close to each other on the same chromosome are likely to be inherited together. The existence of a LD implies that genetic variants will not be distributed independently and could lead to biased results. (3) We computed F-statistics to assess the strength of IVs to make sure these instruments confidently predicted acne status (20). (4) We examined the chosen SNPs one by one on the phenoscanner database and took away IVs that were highly correlated with prostate diseases. (5) We merged the selected IVs with the genetic data of PC acquired from different outcome databases and removed IVs with palindromic sequences, the orientations of which could not be determined, and incompatible SNPs.

### 2.4 Statistical analysis

For MR analysis, the inverse variance weighted (IVW) model was adopted as the main causal evaluation method supplemented by weighted median and weighted MR-Egger, as the IVW method assumed that all IVs were valid (21). Additionally, when directional pleiotropy is absent, the IVW method can deliver a relatively stable and accurate causal evaluation by using a meta-analytic approach to combine Wald estimates for each IV (22).

For sensitivity analysis, the heterogeneity between IVs was tested by Cochrane’s Q-statistic. If significant heterogeneity was indicated, a random-effect model would be adopted. Otherwise, a fixed-effect model would be adopted (23). The MR-Egger intercept method was adopted for detecting the horizontal pleiotropy of SNPs. The MR-Egger intercept test is robustly sensitive to directional bias due to pleiotropy, with a *p*-value of >0.05 indicating the absence of horizontal pleiotropy (24). The leave-one-out sensitivity test was used to judge the stability of the MR results by excluding IVs one by one.

For meta-analysis, the IVW effect estimate as the main evaluation of acne on prostate cancer was acquired from the four outcomes and then combined in a meta-analysis. We adopted a random-effects model to reduce the impact of heterogeneity. Based on the results of the above analyses, we provided an overall evaluation of the genetic causal relationship between acne and prostate cancer.

## 3 Results

### 3.1 Selected IV data

A cohort of 30 IVs was selected from the FinnGen consortium. A cohort of 27 IVs was selected from the UK Biobank. A cohort of 26 IVs was selected from the IEU OpenGWAS project. A cohort of 30 IVs was selected from the European Bioinformatics Institute. All IVs demonstrated strong validity (an F-statistic greater than 10) (**Supplementary Material 1**).

### 3.2 Results of the two-sample MR analysis

We conducted IVW analysis for MR analysis supplemented by MR-Egger and the weighted median. The results of MR analysis from the FinnGen consortium showed a negative causality between acne and PC: IVW [*p*=0.027; odds ratio (OR)=0.925; 95 confidence interval (CI)% 0.863–0.991], MR-Egger (*p*=0.018; OR=0.784; 95 CI% 0.648–0.947), weighted median (*p*=0.114; OR=0.932; 95 CI% 0.857–1.014). The results of MR analysis from the UK Biobank showed no causality between acne and PC: IVW (*p*=0.466; OR=1; 95 CI% 0.999–1.001), MR-Egger (*p*=0.573; OR=1.001; 95 CI% 0.998–1.004), weighted median (*p*=0.394; OR=1; 95 CI% 0.999–1.001). The results of MR analysis from the IEU OpenGWAS project showed no causality between acne and PC: IVW (*p*=0.388; OR=0.978; 95 CI% 0.929–1.029), MR-Egger (*p*=0.903; OR=0.991; 95 CI% 0.853–1.151), weighted median (*p*=0.519; OR=0.981; 95 CI% 0.927–1.039). The results of MR analysis from the European Bioinformatics Institute showed no causality between acne and PC: IVW (*p*=0.563; OR=1.031; 95 CI% 0.931–1.141), MR-Egger (*p*=0.921; OR=1.015; 95 CI% 0.753–1.369), weighted median (P=0.877; OR=1.011; 95 CI% 0.881–1.160) (**Table 2**).

**Table 2:** Results of MR estimates between acne and PC.

Study	Method	SNP (n)	OR (95CI%)	*p*-value
FinnGen consortium	MR-Egger	30	0.784 (0.648–0.947)	0.018
FinnGen consortium	Inverse variance weighted	30	0.925 (0.863–0.991)	0.027
FinnGen consortium	Weighted median	30	0.932 (0.857–1.014)	0.114
UK Biobank	MR-Egger	27	1.001 (0.998–1.004)	0.573
UK Biobank	Inverse variance weighted	27	1 (0.999–1.001)	0.466
UK Biobank	Weighted median	27	1 (0.999–1.001)	0.394
IEU OpenGWAS project	MR-Egger	26	0.991 (0.853–1.151)	0.903
IEU OpenGWAS project	Inverse variance weighted	26	0.978 (0.929–1.029)	0.388
IEU OpenGWAS project	Weighted median	26	0.981 (0.927–1.039)	0.519
European Bioinformatics Institute	MR-Egger	30	1.015 (0.753–1.369)	0.921
European Bioinformatics Institute	Inverse variance weighted	30	1.031 (0.931–1.141)	0.563
European Bioinformatics Institute	Weighted median	30	1.011 (0.881–1.160)	0.877

### 3.3 Results of the sensitivity analysis

**Figure 2:**
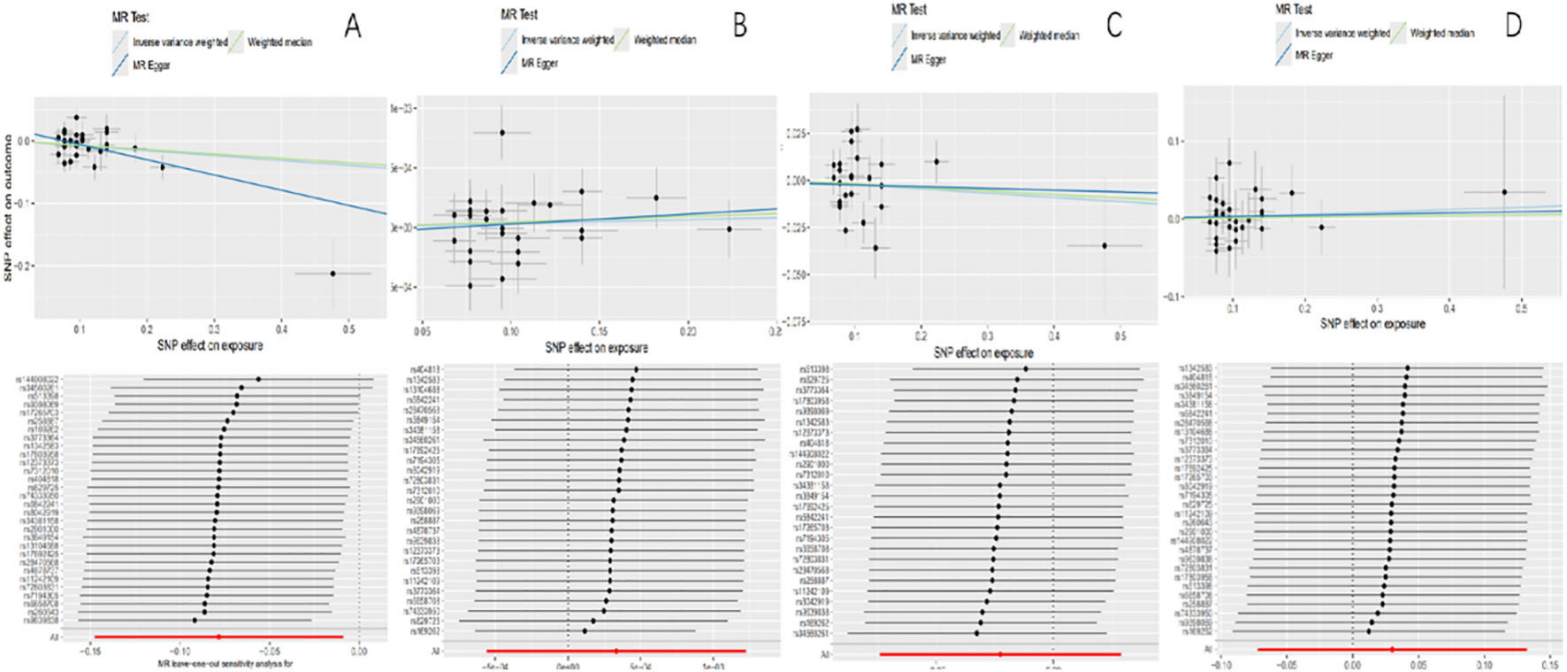
Scatter plots of causality and “leave-one-out” sensitivity tests. **(A)** The FinnGen consortium. **(B)** The UK Biobank. **(C)** The IEU OpenGWAS project. **(D)** The European Bioinformatics Institute.

The values of intercept were all small, and horizontal pleiotropy was not significant (*p*_Finn_=0.078, *p*_UKB_=0.718, *p*_IEU_=0.855, *p*_EBI_=0.918). The results of Cochran’s Q test of MR exploration (*p*_Finn_=0.0216, *p*_UKB_=0.072, *p*_IEU_=0.001, *p*_EBI_=0.895) indicated significant heterogeneity. However, it did not affect the accuracy of the result as a multiplicative random-effects model was adopted in this MR analysis. No single SNP was seen in a leave-one-out test that had a disproportionate effect on the overall results (**Table 3**; **Figure 2**).

**Table 3:** Results of the sensitivity analysis.

Study	Pleiotropy		Heterogeneity	
	Intercept	*p*-value	Q	*p*-value
FinnGen Consortium	0.019	0.078	46.36	0.0216
UK Biobank	<0.001	0.718	37.17	0.072
IEU OpenGWAS project	-0.001	0.855	51.41	0.001
European Bioinformatics Institute	0.001	0.918	20.99	0.859

### 3.4 Results of the meta-analysis

**Figure 3:**
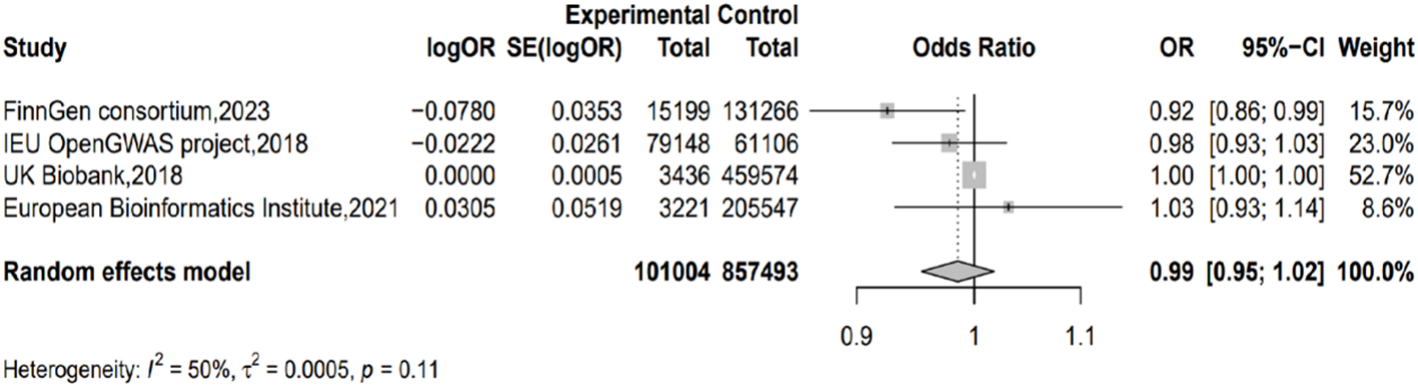
Forest plot of the results from the MR analysis.

Taking IVW as the major method, we conducted meta-analysis on the results from different outcome databases. The result did not show significant heterogeneity (I^2^ = 49.6%; H=1.41; *p*=0.11) or a genetic causal relationship between acne and PC (*p*=0.378; OR=0.985; 95% CI 0.954–1.018) (**Figure 3**).

## 4 Discussion

To the best of our knowledge, this is the first study investigating the association between acne and PC that applied MR analysis. Additionally, we integrate the MR results from different outcome databases to draw a reasonable conclusion. Finally, the result supports there is no genetic causal relationship between acne and PC.

Until now, the relationship between acne and the risk of PC has remained unclarified. Previous studies reported inconsistent results. A large prospective population‐based cohort suggested that a diagnosis of acne classified as severe conferred a sixfold increased risk of PC (11). Another prospective cohort study suggested that acne during young adulthood was associated with an increased risk of PC‐specific death (12). A prospective cohort of American male health professionals showed an increased PC risk for men who reported receiving treatment with tetracycline for more than 4 years, which was regarded as a marker of severe acne (25). Additionally, there were studies that showed no association between acne and the risk of PC (14, 15). There was still a case-control study that reported a negative association between acne‐related facial scarring and later PC (16). However, most of these studies are observational studies. Furthermore, these contentious findings may be influenced by indissoluble or unidentified risk factors and recall bias.

The exact pathogenic mechanism of PC is still largely unknown. There have been two different hypotheses regarding the association between acne and PC so far. The first is that *P. acnes*-mediated inflammation may contribute to PC. A study (26) found that *P. acnes* isolated from radical prostatectomy specimens was positively related to the onset and extent of both acute and chronic prostate inflammation. Persistent low-grade *P. acnes*-mediated inflammation may lead to precursor lesions for PC (25). The second is a strong immune response caused by *P. acnes* may offer protection from PC. *P. acnes* can cause inflammatory reactions through complement activation and the induction of proinflammatory cytokines (27) and can also cause a strong cell-mediated T-helper type 1 (Th1) immune response (28). Additionally, Th1 immune responses are detrimental to the establishment and progression of tumors (29). One study (30) reported that high serum titers of antibodies directed against *P. acnes* were observed to be inversely associated with the risk of PC, and the high serum titers of antibodies directed against *P. acnes* may be an indirect marker of increased cell-mediated immunity. One of our MR results pointed to a similar conclusion. However, MR analysis of a population only provides a quasi-randomized controlled trial design. The findings require validation in different populations. Additionally, hormonal activity also plays a role in the pathology of acne. In previous studies, the association between *P. acnes* and PC could not fully represent the association between acne and PC. Our study integrating MR results from different populations suggests acne should not be considered as a morbidity hazard factor for PC. However, the effects of *P. acnes* on PC still need further research.

There are several strengths in our study. First, to our knowledge, this is the first study using MR analysis to investigate the association between acne and PC. MR studies can effectively avoid confounding bias compared with observational studies. Second, a supplementary meta-analysis and various sensitivity tests were implemented to increase the reliability of the results. Third, the study may help to influence public health policies regarding the early prevention and timely intervention of PC.

There are still some limitations in our study. First, the age discrepancy between acne and PC could introduce bias. Genetic variants for acne may not remain relevant or active during the typical onset age of PC. Age-stratified analyses could mitigate this. However, current GWAS data on acne lack more detailed age information, limiting the possibility of conducting subgroup analysis. Second, the GWAS data came from the European population. Whether our described findings would be consistent in other populations remained to be investigated. Genetic data from more numerous and larger-scale GWASs are needed. Third, the concrete mechanism of PC is still unclear. Interrelationships between *P. acnes*, other prostate diseases, and PC still need further research.

## 5 Conclusion

Our MR combined with meta-analysis suggests there is no genetic causal relationship between acne and PC. Acne should not be considered as a morbidity hazard factor for PC.

## Data Availability

Publicly available datasets were analyzed in this study. These data can be found from FinnGen consortium (https://r10.finngen.fi/), UK Biobank (https://www.ukbiobank.ac.uk/) EuropeanBioinformatics Institute (https://www.ebi.ac.uk/gwas/), and IEUOpenGWAS project (https://gwas.mrcieu.ac.uk/).
